# Supplementary material for: Conservation of a flagship species: Health assessment of the pink land iguana, Conolophus marthae
Source: PLoS One. 2022 Mar 29;17(3):e0257179. doi: 10.1371/journal.pone.0257179 (PMC8963547; doi:10.1371/journal.pone.0257179)
Supplement: S1 Fig — (DOCX) [file pone.0257179.s001.docx]

S1 Figure


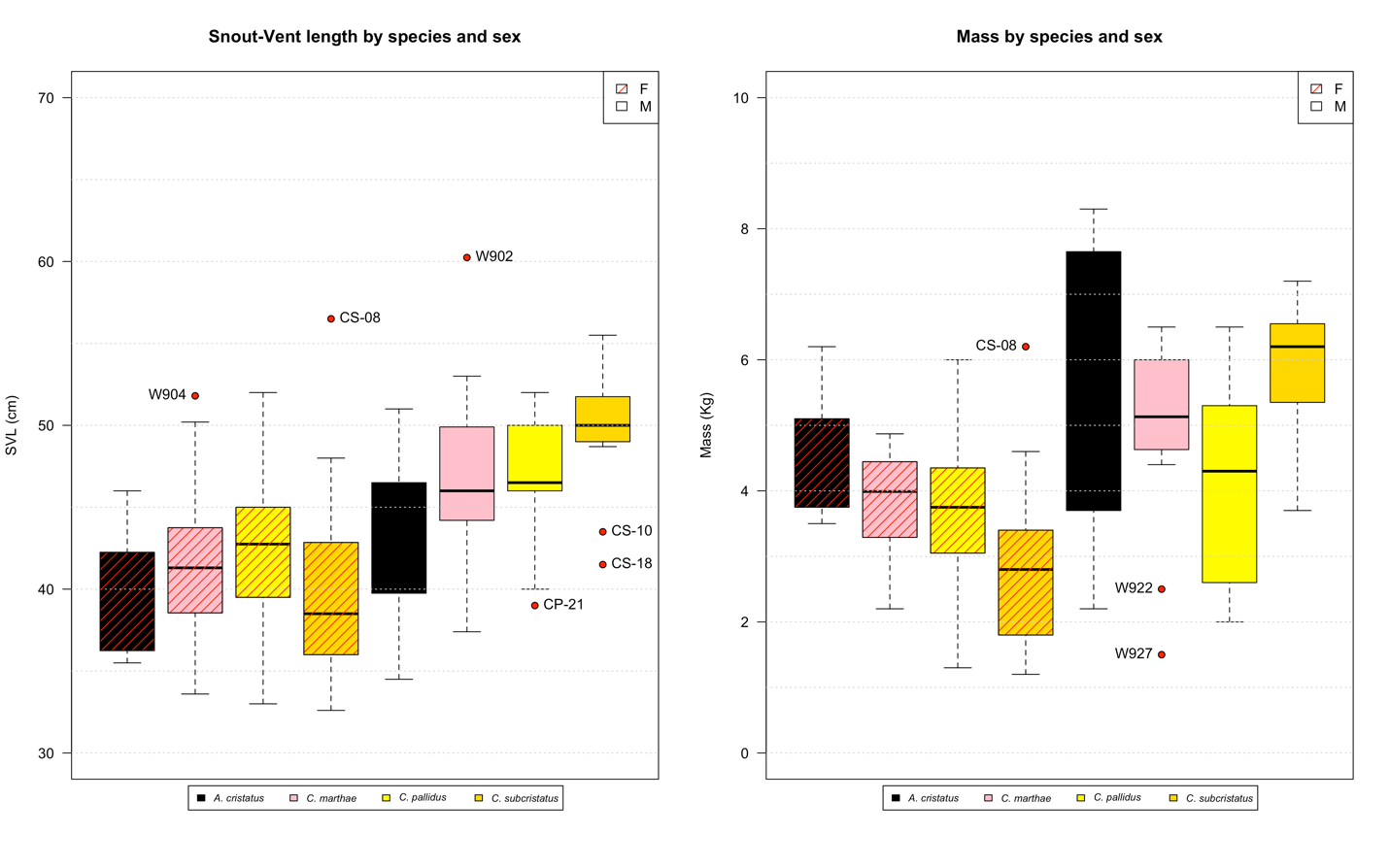


Identification of outlier values in morphological features. Red dots represent individuals for which outlier values have been identified. The text next to each outlier point refers to a unique individual identifier.
