# Supplementary figures and images for: Conservation of a flagship species: Health assessment of the pink land iguana, Conolophus marthae
Source: PLoS One. 2022 Mar 29;17(3):e0257179. doi: 10.1371/journal.pone.0257179 (PMC8963547; doi:10.1371/journal.pone.0257179)

S2 Figure


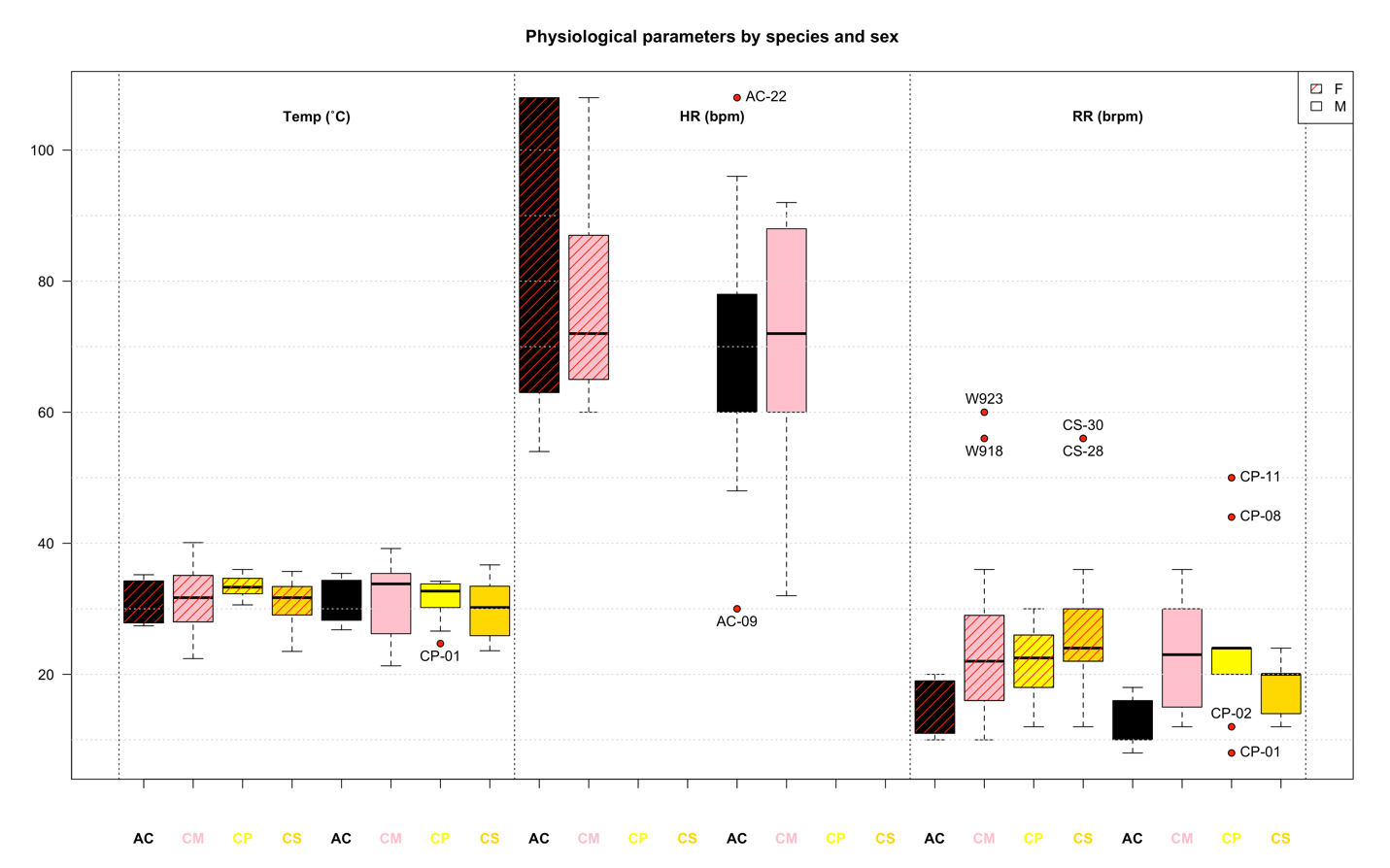

Supplement: S2 Fig — (DOCX) [file pone.0257179.s002.docx]

S3 Figure


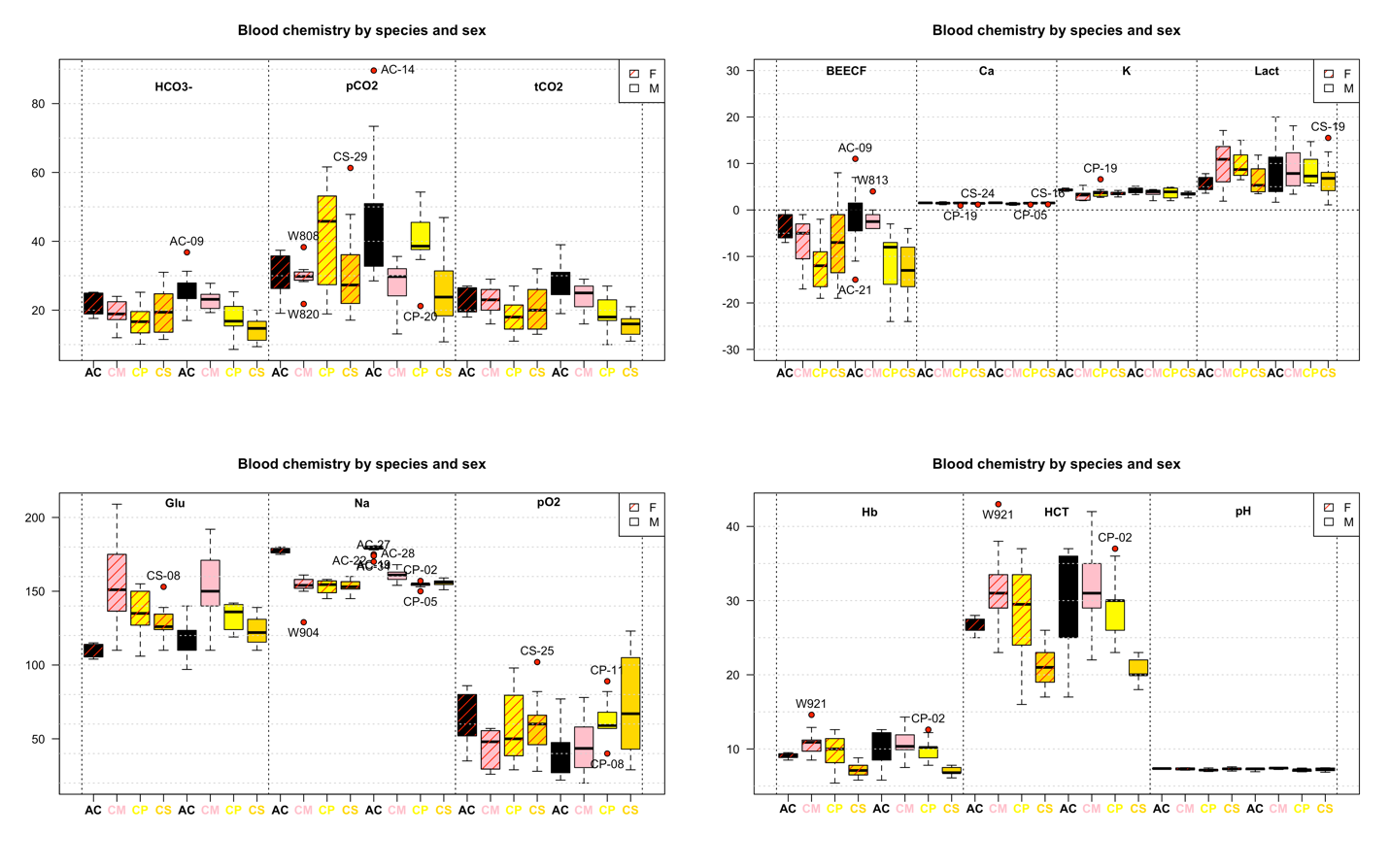

Supplement: S3 Fig — (DOCX) [file pone.0257179.s003.docx]

S4 Figure


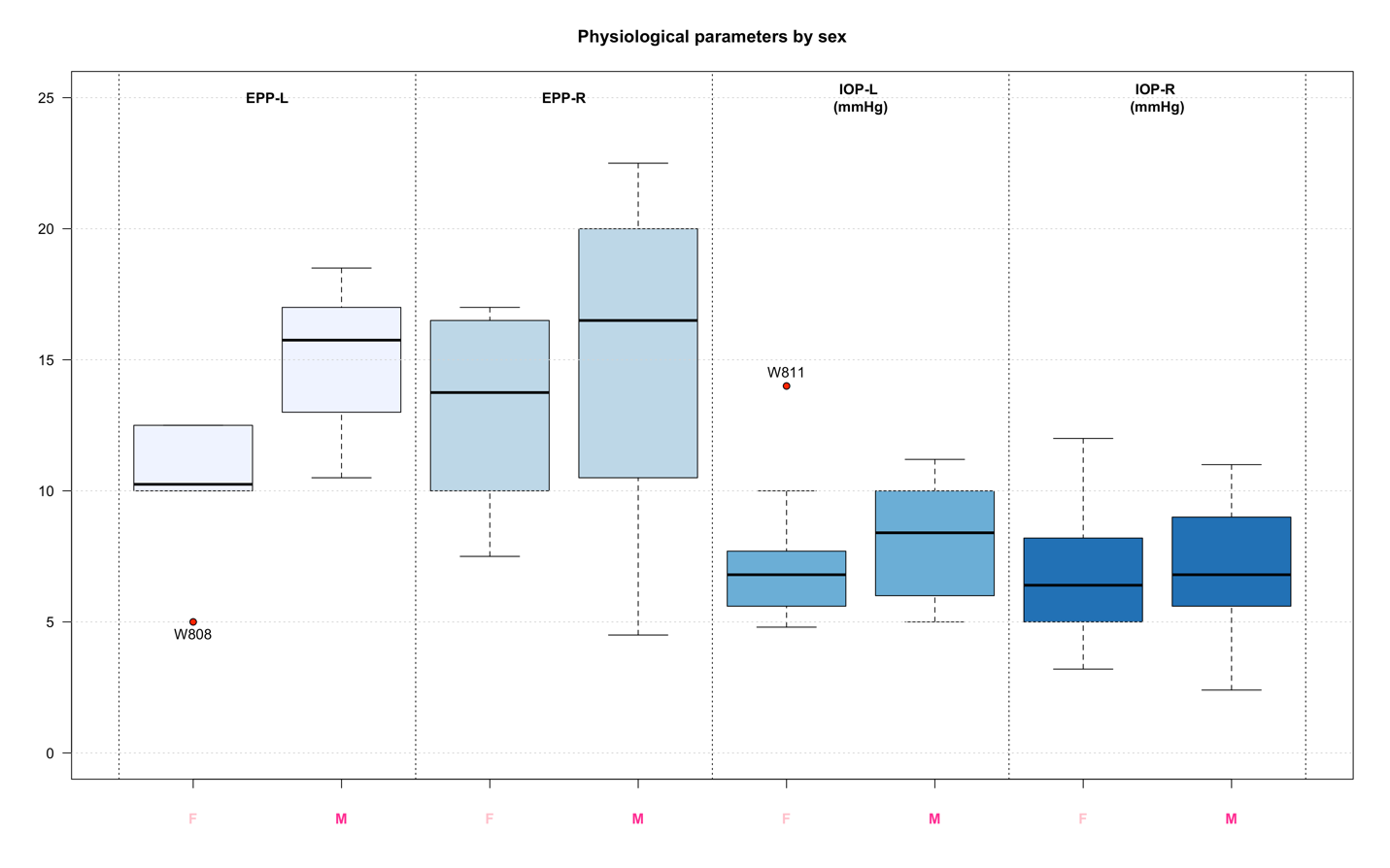

Supplement: S4 Fig — (DOCX) [file pone.0257179.s004.docx]
