# Supplementary material for: Conservation of a flagship species: Health assessment of the pink land iguana, Conolophus marthae
Source: PLoS One. 2022 Mar 29;17(3):e0257179. doi: 10.1371/journal.pone.0257179 (PMC8963547; doi:10.1371/journal.pone.0257179)
Supplement: S5 Fig — (DOCX) [file pone.0257179.s005.docx]

S5 Figure


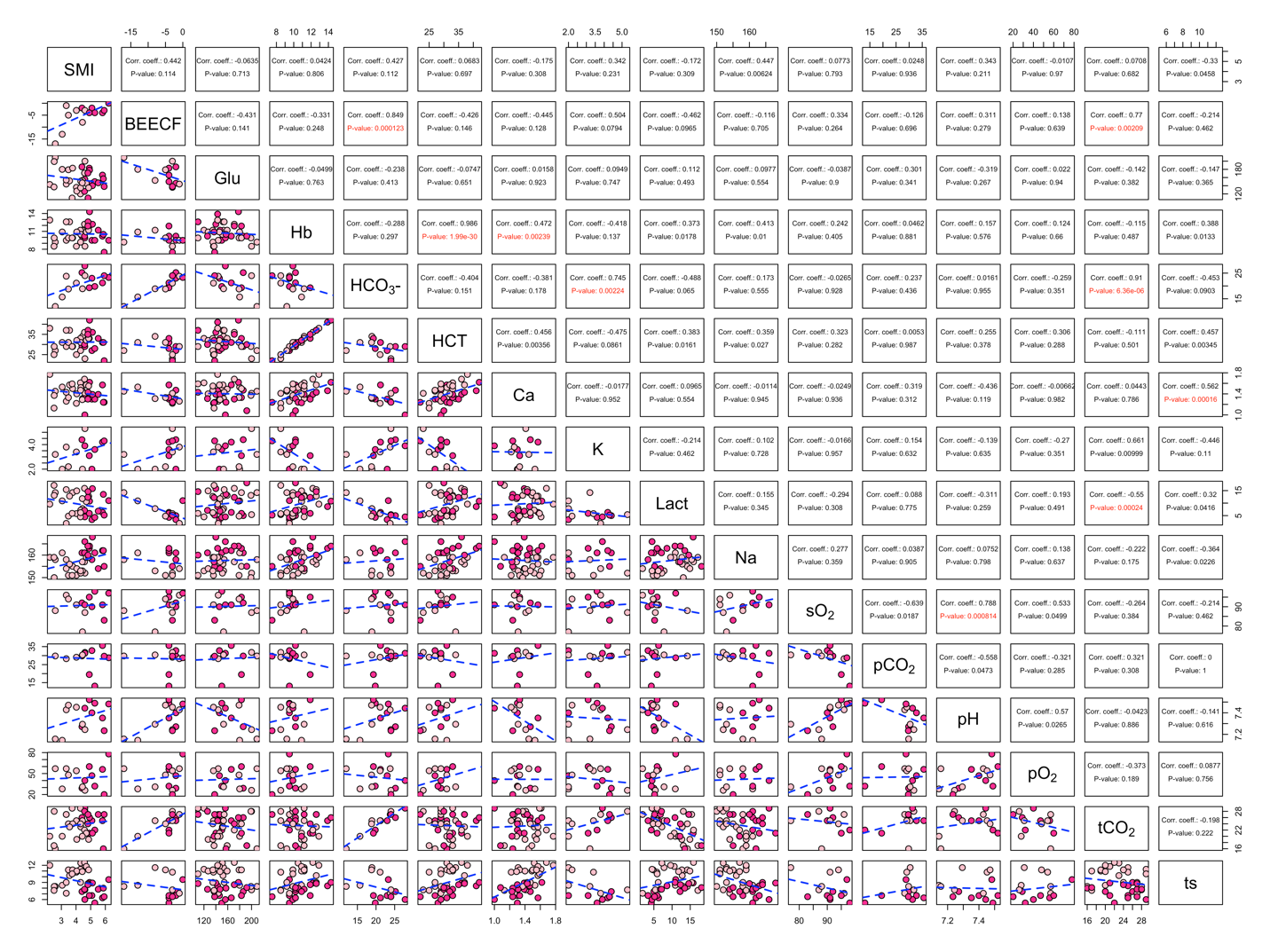


Spearman’s Rank Correlation values estimated on linear regression lines between variables listed across the diagonal. Significant correlations are highlighted in red.
