# Supplementary material for: Conservation of a flagship species: Health assessment of the pink land iguana, Conolophus marthae
Source: PLoS One. 2022 Mar 29;17(3):e0257179. doi: 10.1371/journal.pone.0257179 (PMC8963547; doi:10.1371/journal.pone.0257179)
Supplement: S1 Table — (DOCX) [file pone.0257179.s006.docx]

**S1 Table Comparison of measurements using different i-Stat cartridges**

|  | | Chem8 | | | | CG8 | | | | P |
| --- | --- | --- | --- | --- | --- | --- | --- | --- | --- | --- |
|  |  | Mean | Sd | Min/Max | N | Mean | Sd | Min/Max | N |  |
| Males | Glu | 160.71 | 18.41 | 138/186 | 7 | 155.88 | 16.29 | 138/185 | 8 | 0.68 |
|  | Ca | 1.32 | 0.18 | 1/1.57 | 7 | 1.36 | 0.17 | 1.04/1.56 | 8 | 0.77 |
|  | Na | 160.86 | 3.53 | 155/166 | 7 | 162.00 | 4.54 | 154/168 | 8 | 0.48 |
|  | K | 3.64 | 0.85 | 2/4.4 | 7 | 3.64 | 0.85 | 2/4.4 | 7 | 0.18 |
|  | Hct | 28.43 | 3.46 | 22/33 | 7 | 28.75 | 3.58 | 22/35 | 8 | 0.95 |
| Females | Glu | 171.57 | 28.01 | 136/209 | 7 | 167.14 | 30.53 | 131/212 | 7 | 0.60 |
|  | Ca | 1.36 | 0.11 | 1.22/1.53 | 7 | 1.41 | 0.13 | 1.22/1.53 | 7 | 0.56 |
|  | Na | 154.29 | 3.82 | 151/161 | 7 | 153.71 | 5.02 | 149/162 | 7 | 0.69 |
|  | K | 3.13 | 1.20 | 2/5.3 | 7 | 3.13 | 1.20 | 2/5.3 | 7 | 0.65 |
|  | Hct | 28.57 | 3.82 | 23/34 | 7 | 28.43 | 3.15 | 25/33 | 7 | 0.99 |

This table shows the comparison between blood chemical values recorded using different i-Stat cartridges (Chem8 and CG8) in males and females. Last column (P) shows the P-value of Wilcoxon Rank Sum Test comparison.
